# *N-Myc* promotes angiogenesis and therapeutic resistance of prostate cancer by *TEM8*

**DOI:** 10.1007/s12032-021-01575-x

**Published:** 2021-09-14

**Authors:** Mingfeng Li, Linna Fang, Louis Boafo Kwantwi, Guifang He, Wenwu Luo, Lijie Yang, Yuhang Huang, Shiyuan Yin, Yongping Cai, Wei Ma, Heqin Zhan, Zhuting Tong, Li Zhang, Chaozhao Liang, Yu Yin

**Affiliations:** 1 Department of Pathology, Anhui Medical University, 81 Meishan Road, Hefei, 230032 Anhui People’s Republic of China; 2 Department of Pathology, First Affiliated Hospital of Anhui Medical University, Hefei, 230022 Anhui People’s Republic of China; 3 Department of Radiotherapy, First Affiliated Hospital of Anhui Medical University, Hefei, 230022 Anhui People’s Republic of China; 4 Department of Urology, First Affiliated Hospital of Anhui Medical University, 218 Jixi Road, Hefei, 230022 Anhui People’s Republic of China

**Keywords:** Prostate cancer, *N-Myc*, *TEM8*, Angiogenesis, ADT

## Abstract

Although patients with early localized prostate cancer can survive longer, castration-resistant prostate cancer (CRPC) has gradually emerged with the use of androgen deprivation therapy (ADT). *N-Myc* and *TEM8* play a vital role in the progression of several cancer types. However, the underlying mechanism of how *N-Myc* and *TEM8* promote the progression of prostate cancer remains unclear. In this study, the expression of *N-Myc* and *TEM8* was detected in benign prostatic hyperplasia (BPH) and prostate cancer (PCa) tissues by immunohistochemistry (IHC). LNCaP cell lines were maintained in RPMI 1640 medium supplemented with 10% charcoal-stripped fetal bovine serum. Subsequently, R language software was used to verify our results. Tubule formation assay of human umbilical vein endothelial cell (HUVEC) was conducted to examine the effect of *N-Myc* and *TEM8* overexpression on angiogenesis in prostate cancer cells. IHC results showed a positive correlation between the expression of *N-Myc* and *TEM8* in prostate cancer tissues. Further analysis showed that *N-Myc* and *TEM8* were associated with clinicopathological features and poor prognosis in prostate cancer patients. Moreover, the overexpression of *N-Myc* and *TEM8* promoted proliferation of prostate cancer cells and angiogenesis. Additionally, *N-Myc* and *TEM8* overexpression was associated with therapeutic resistance. We further found that N*-Myc* promoted angiogenesis and therapeutic resistance in prostate cancer via *TEM8*. Hence, targeting *N-Myc*/*TEM8* pathway in prostate cancer would be a novel therapeutic strategy to enhance the treatment of prostate cancer patients.

**Supplementary Information:**

The online version contains supplementary material available at 10.1007/s12032-021-01575-x .

## Introduction

Prostate cancer (PCa) is the most diagnosed malignancy in men and the second leading cause of cancer-related deaths in the USA. Although the recent improvement in treatment options has significantly reduced the incidence rate, there has been a steady increase in advanced or metastasized prostate cancers [1]. Generally, the symptoms of prostate cancer in the early stage are not evident in most cases, which makes treatment more difficult [2]. Prostate cancer progression follows a multistep process that includes prostate intraepithelial neoplasia (PIN), local prostate cancer, advanced prostate adenocarcinoma with local invasion, and metastatic prostate cancer [3]. Recently, the advent usage of androgen deprivation therapy (ADT) has brought some symptomatic relief to prostate cancer patients. However, this also leads to the development of castration-resistant prostate cancer. Therefore, a deeper understanding of the mechanism of the disease process is relevant in our quest to find a novel and specific biomarker for prostate cancer treatment.

*N-Myc*, a member of the *MYC* proto-oncogene family, is known to play several oncogenic activities. Mounting studies have indicated that *N-Myc* is involved in all facets of prostate cancer progression [4–6], including the transformation of castration-resistant prostate cancer to neuroendocrine [7, 8]. Previous study has shown that *TEM8* is upregulated in LNCaP and 22RV1 prostate cancer cell lines, particularly after the overexpression of *N-Myc* [7]. However, the detailed mechanism of how *N-Myc* interacts with *TEM8* to promote prostate cancer progression remains unknown. *TEM8*, also known as *ANTXR1*, is a cell-surface transmembrane protein initially identified in the vascular endothelial cells of colon cancer [9]. Some studies have shown that the vascular density in high-grade prostate cancer is significantly higher than that in low-grade prostate cancer tumors [10], which implicates angiogenesis as a mechanism of prostate cancer progression. Even though *TEM8* is associated with poor prognosis in several solid tumors [11–14], little is known about its role in prostate cancer progression. Therefore, this study was designed to elucidate how *N-Myc* interacts with *TEM8* to promote angiogenesis and treatment resistance in prostate cancer.

## Materials and methods

### Patients and specimens

Formalin-fixed paraffin-embedded tissues were collected from 151 patients who underwent surgical operation at the First Affiliated Hospital of Anhui Medical University. The protocols used in the study were approved by the ethical committee of Anhui medical university.

### RT-qPCR assays

Total RNA was isolated from cultured cells using Trizol reagent (Invitrogen). RNA was reversely transcribed into cDNA using the PrimeScript RT Master Mix (Takara, RR036A). Gene-specific primers are as follows:

*GAPDH*: Forward, 5′-CATGAGAAGTATGACAACAGCCT-3′,

Reverse, 5′-AGTCCTTCCACGATACCAAAGT-3′;

*N-Myc*: Forward, 5′-CACGTCCGCTCAAGAGTGTC-3′,

Reverse, 5′-GTTTCTGCGACGCTCACTGT-3′;

*TEM8*: Forward 5′-GATGATGATGGTCTGCCTAAGA-3′,

Reverse 5′-TCTTTGCCTTTTCCAACTTAGC-3′.

### Cell culture and construction

The cell lines were maintained in RPMI 1640 medium (HyClone, SH30809.01) supplemented with 10% FBS (BI, 04-001-01A) or 10% charcoal-stripped fetal bovine serum (MRC, CCS30010.01HT,), 1.5 mM L-Glutamine, and 1% penicillin–streptomycin solution. Lentiviral vector for *N-Myc* and *TEM8* was purchased from Genepharma (Shanghai, China) and stably transfected into LNCap and C4-2 cell lines. Finally, stable prostate cancer cells were successfully screened with the appropriate concentration of puromycin. The specific concentration of puromycin used in screening LNCaP stable cell lines is 2.00ug/ml, and the concentration of puromycin used to screen C4-2 is 1.00ug/ml. The efficiency of overexpression was analyzed by quantitative real-time PCR (qPCR) and western blot. Also, cell lines with *N-Myc* overexpression were transfected with *shRNA-TEM8*. The sequences of *shRNA-TEM8* were designed as follows: GCTGAACCATCCACCATATGT. A non-targeting shRNA (Genepharma) was used as a control.

### Western blotting

Proteins were extracted from prostate cancer cells using RIPA buffer. Proteins were loaded onto 10% SDS-PAGE gels and then transferred to NC membranes. Nonspecific binding was blocked with 5% dried skim milk. The membranes were incubated overnight at 4 ℃ with the following primary antibodies: anti-*GAPDH* (dilution 1:1000, ProteinTech, 10,494-1-AP), anti-*N-Myc* (dilution 1:1000, CST, #51705 s), anti-*TEM8* (dilution 1:400, Abcam, #13,798), and anti-*AR* (dilution 1:2000, CST, 5153 s). After incubation with a peroxidase-conjugated secondary antibody, the protein bands were observed with ECL. The intensity of the protein bands was normalized with *GAPDH*.

### Immunohistochemical analysis

Formalin-fixed paraffin-embedded samples were obtained from benign prostatic hyperplasia and prostate cancer patients. Briefly, sections were deparaffinized with xylene and rehydrated with graded ethanol. Heat-induced antigen retrieval was performed according to the relevant antibody instructions and endogenous peroxidase activities were blocked with hydrogen peroxide for 10 min at room temperature. The sections were incubated with the following antibodies: anti-*N-Myc* antibody (dilution 1:640, CST, #51705 s), anti-*TEM8* antibody (dilution 1:20, NOVUS, 200C1339). The scoring of *N-Myc* and *TEM8* was performed as previously described [15].

### Cell counting Kit-8

100ul of cell suspension with a concentration of 3 × 10^4^ /ml was added to each well (96-well plate) and cultured for 1 day, 2 days, and 3 days. After incubation with 10 ul of cck8 solution for 4 h, the absorbance was measured at 450 nm. The fold change of cell viability was equal to 100% × (As–Ab)/(Ac−Ab): As = OD450nm of the experimental group; Ab = OD450nm of the blank control; Ac = OD450nm of the control.

### Tubule formation assay

Matrigel (# 356,234, Corning) was added into precooled 96-well plates (60μL/well) and incubated at 37 °C for 30 min. 3 × 10^4^ HUVEC cells were inoculated into each well, and the corresponding concentrated supernatant was added and placed into the incubator for further incubation. The tube formation was observed under an inverted microscope at different time points. ImageJ was used to measure the length of the tube.

### Oncomine and GEO datasets analysis

Oncomine microarray database (https://www.oncomine.org) was used to validate the prognostic value of *N-Myc* and *TEM8* in prostate cancer patients. In this study, the threshold was defined as *p*-value = 0.0001. The overall survival and expression levels of *N-Myc* and *TEM8* were plotted using GraphPad Prism software.

In GSE150368 datasets, the LIMMA package and Edger package of R software was used to detect and screen differentially expressed genes (DEGs) in prostate tissue before and after ADT treatment. DEGs analysis was performed based on the screening criteria of FDR > 0.05 and |log (FC)|> 1. KEGG enrichment analysis was used to detect the relationship between the function and signaling pathway of DEGs. Furthermore, the String Online database (confidence score of ≥ 0.15) was used to identify the protein–protein interaction (PPI) network (https://string-db.org/).

### Statistical methods

All the experiments had three independent replicates, and the data were presented as mean ± SD. Statistical analyses were performed using Pearson chi-square test or Log-rank test unless otherwise indicated. A *p*-value of < 0.05 was considered statistically significant.

## Results

### *N-Myc* and *TEM8* expression were associated with clinicopathological features of PCa and there was a positive correlation between them

**Fig. 1:**
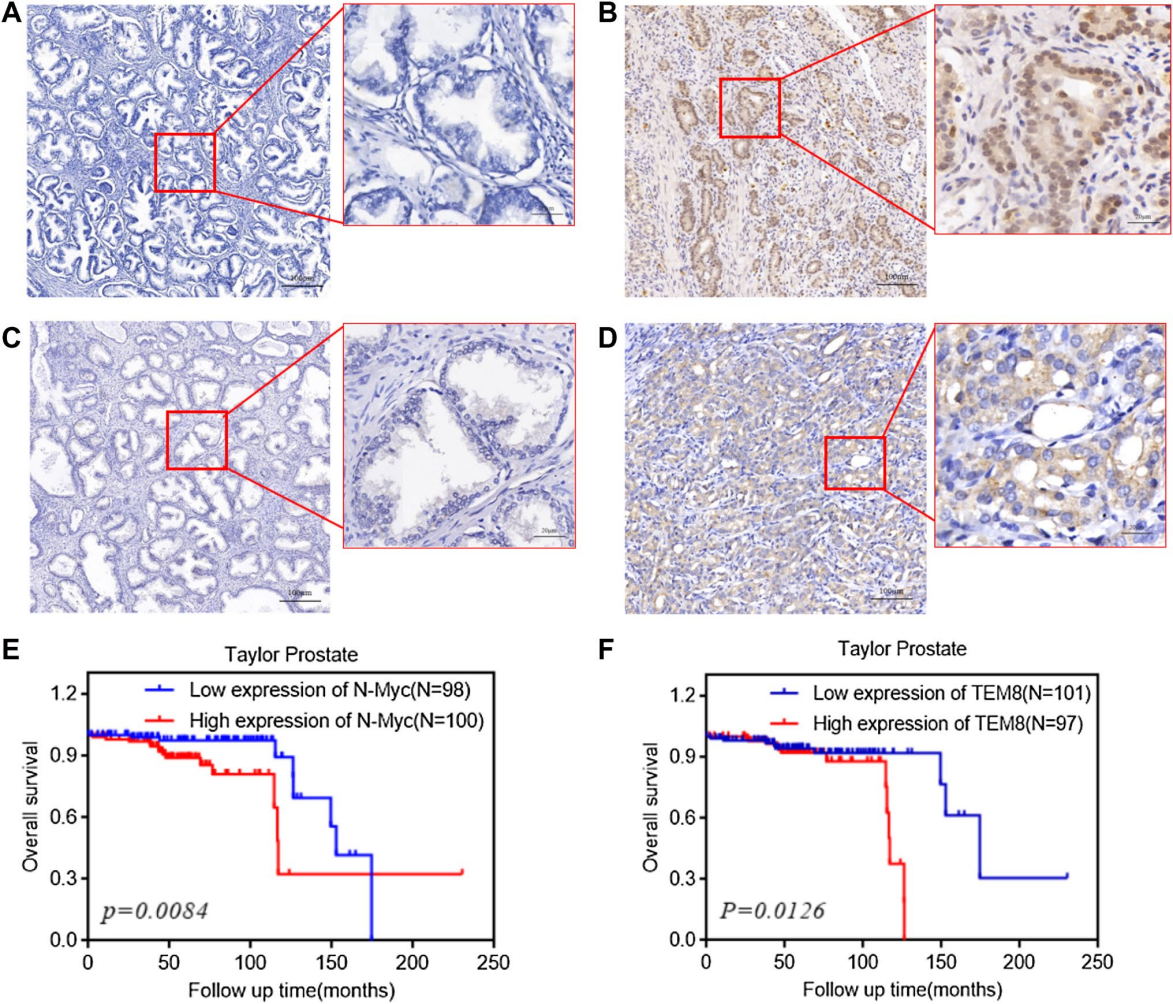
Expression of *N-Myc* and *TEM8* in prostate tissues and their correlation with prognosis. **A** *N-Myc* was negatively expressed in BPH. **B** *N-Myc* was positively expressed in PCa (left), and *N-Myc* was expressed in the nucleus and cytoplasm of PCa cells (right). **C** *TEM8* was negatively expressed in BPH. **D** *TEM8* was positively expressed in PCa (left), and *TEM8* was expressed in the membrane and/or cytoplasm of PCa cells (right). (The magnification of Fig. 1. **A**–**D** is 100X on the left and 400X on the right). (E, F) *N-Myc* and *TEM8* were related to the overall survival rate of PCa as shown by Taylor Prostate dataset in the Oncomine database

IHC results showed that the positive rate of *N-Myc* and *TEM8* was significantly higher in PCa than in BPH samples (Fig. 1A–D; Table 1). High expressions *N-Myc* and *TEM8* were associated with a high Gleason score, advanced TNM stage, and osseous metastasis. However, *TEM8* but not *N-Myc* was associated with elevated PSA levels (Table 2).

**Table 1:** The expression of *N-Myc*, *TEM8* in prostate clinical samples

Group	*n*	*N-Myc*		*p-value*	*TEM8*		*p-value*
		Low,*n*(%)	High,*n*(%)		Low,*n*(%)	High,*n*(%)	
Benign Prostate	60	56(93.33)	4(6.67)	0.002^*^	57(95.00)	3(5.00)	< 0.001^***^
Adenocarcinoma	91	67(73.63)	24(26.37)		33(36.26)	58(63.74)	

*P < 0.05, ***P < 0.001

**Table 2:** Clinicopathological parameters in prostate cancer

Characteristic	*n*	*N-Myc* expression		*P -value*	*TEM8* expression		*P -value*
		Low,*n*(%)	High,*n*(%)		Low,*n*(%)	High,*n*(%)	
Age, years							
≤ 70	45	30(66.67)	15(33.33)	0.136	18(40.00)	27(60.00)	0.463
> 70	46	37(80.43)	9(19.57)		15(32.61)	31(67.39)	
PSA at initial diagnosis (mg/L)							
< 20	40	32(80.49)	8(19.51)	0.222	20(50.00)	20(50.00)	0.016*
≥ 20	51	35(68.00)	16(32.00)		13(25.49)	38(74.51)	
Gleason score							
≤ 7	43	38(88.37)	5(11.63)	0.003**	21(48.84)	22(51.16)	0.018*
> 7	48	29(60.42)	19(39.58)		12(25.00)	36(75.00)	
TNM stage							
I–II	45	40(88.11)	5(11.11)	0.001**	22(48.89)	23(51.11)	0.013*
III–IV	46	27(58.70)	19(41.30)		11(23.91)	35(76.09)	
Osseous metastasis							
No	71	59(83.10)	12(16.90)	< 0.001***	30(42.25)	41(57.75)	0.025*
Yes	20	8(40.00)	12(60.00)		3(15.00)	17(85.00)	

*P < 0.05, **P < 0.01, ***P < 0.001

We observed that *N-Myc* was positively correlated to the expression of *TEM8* in PCa (R = 0.244, *P* = 0.02) (Table 3). According to the oncomine database, patients with high expression of *N-Myc* or *TEM8* had a significantly lower overall survival rate than those with low *N-Myc* or *TEM8* expression (Fig. 1E, F). Taken together, *N-Myc* and *TEM8* expressions are closely related to the clinical progression and prognosis of prostate cancer.

**Table 3:** Correlation between expression of *N-Myc* and *TEM8* in prostate cancer tissues

*N-Myc*	*TEM8*		*n*	*r*_*s*_	*P -value*
	High	Low			
High	20	4	24	0.244	0.02*
Low	38	29	67		
*n*	58	33	91		

*P < 0.05

### *N-Myc* overexpression upregulated *TEM8* expression in PCa cells

**Fig. 2:**
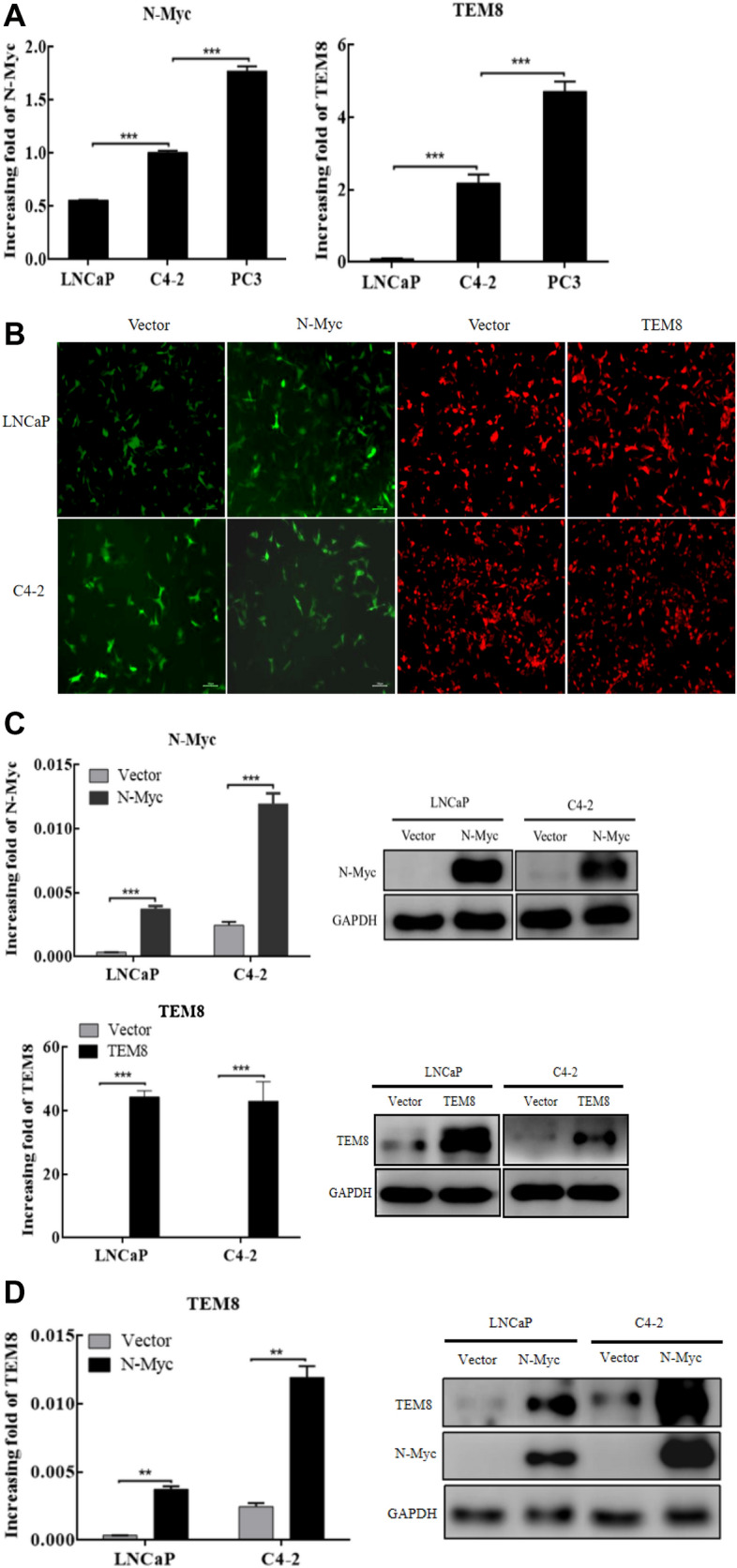
Overexpression of *N-Myc* and *TEM8* using lentivirus in prostate cancer cell lines. **A** The mRNA expression level of *N-Myc* and *TEM8* in LNCaP, C4-2, and PC3 cell lines. **B** *N-Myc* and *TEM8* overexpressing stable cell lines for LNCaP and C4-2 by lentivirus infection as observed under fluorescence microscope (× 100). **C** Expression of *N-Myc* and *TEM8* were detected in lentivirus-transfected prostate cell lines at mRNA and protein levels. **D** Expression of *TEM8* was detected in *N-Myc* overexpressing stable cell lines (*P < 0.05, **P < 0.005, ***P < 0.001)

This study found that high expression of *N-Myc* and *TEM8* was associated with high degree of prostate cancer (*P* < 0.05) (Fig. 2A), suggesting that *N-Myc* and *TEM8* are involved in the progression of prostate cancer. To further study the relationship between *N-Myc* and *TEM8* in prostate cancer progression, stable cell lines with *N-Myc* and *TEM8* overexpression were generated by lentivirus infection. The results were observed by fluorescence microscopy (Fig. 2B). Subsequently, the overexpression of *N-Myc* and *TEM8* was verified in LNCaP and C4-2 cell lines by western blot and PCR (Fig. 2C).

To validate the results obtained from clinical samples, the mRNA and protein expressions of *TEM8* were detected in *N-Myc* overexpressing stable cell lines. Our study further confirmed that *N-Myc* regulated the expression of *TEM8* in prostate cancer cells (Fig. 2D).

### Overexpression of *N-Myc* and *TEM8* promoted the proliferation and tubule formation in prostate cancer cells

**Fig. 3:**
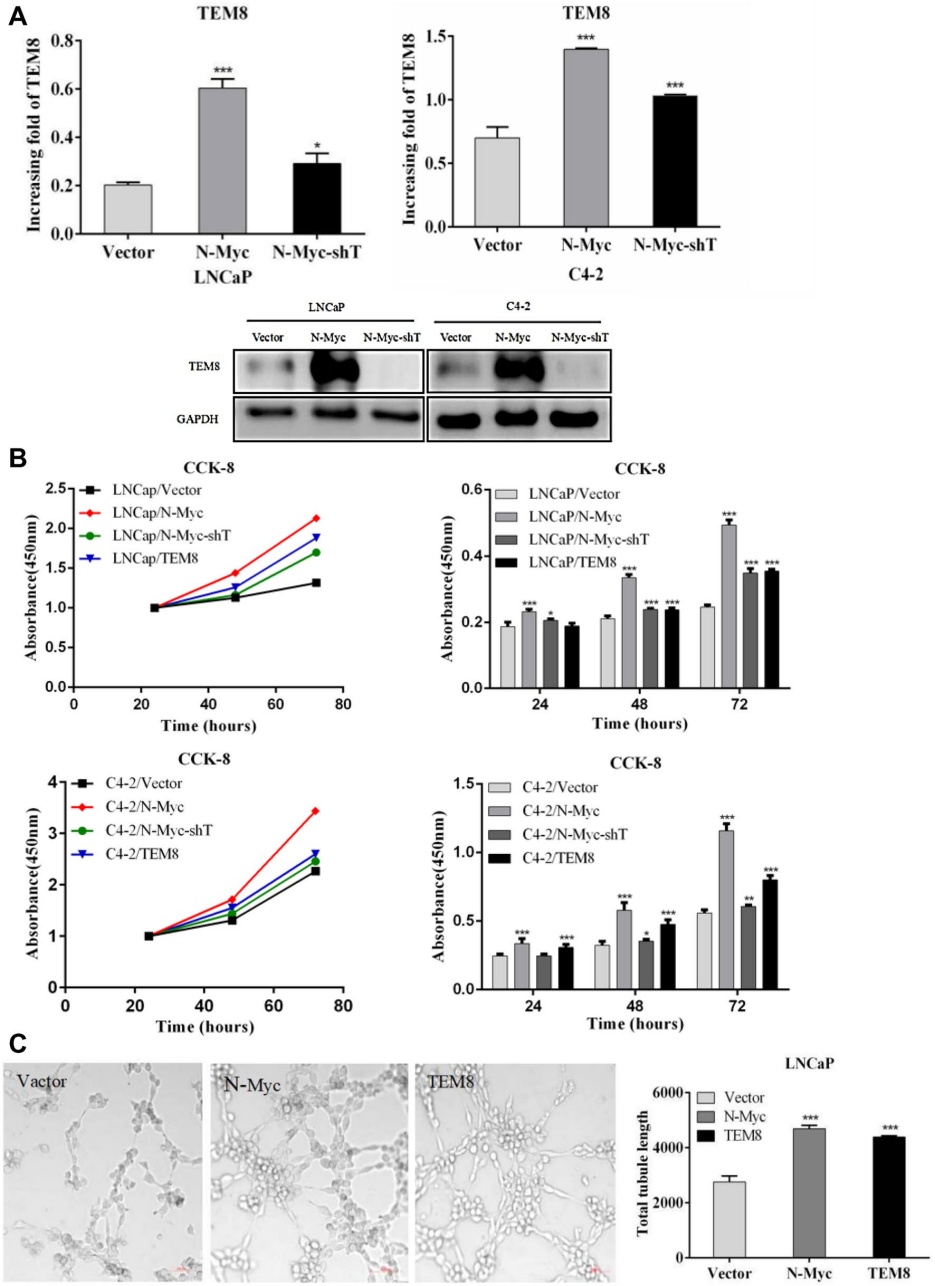
Effects of *N-Myc* and *TEM8* on the proliferative ability and tubule formation of PCa cells. **A** The mRNA and protein expressions of *TEM8* were verified after LNCaP/*N-Myc* and C4-2/*N-Myc* cells were treated with *TEM8*-shRNA. **B** Compared with the control group, the overexpression of *N-Myc* and *TEM8* affected the proliferative ability of LNCaP and C4-2 cells. **C** HUVEC tube formation assay was determined using the supernatant from LNCaP cells

In *N-Myc* overexpressing stable cells (LNCaP and C4-2), the expression of *TEM8* was verified after *TEM8* shRNA knockdown (Fig. 3A). Compared with LNCaP/Vector, the growth rate of cancer cells was significantly higher in LNCaP/*N-Myc* and LNCaP/*TEM8* groups (*P* < 0.05). However, the proliferation of cancer cells was lower in LNCaP/*N-Myc*/*shTEM8* than LNCaP/*N-Myc*. A similar trend was observed in the C4-2 group (Fig. 3B). Next, we found from tube formation experiments that overexpression of *N-Myc* and *TEM8* significantly promoted angiogenesis in prostate cancer (Fig. 3C).

### *N-Myc* overexpression confers LNCaP cells resistant to ADT treatment

**Fig. 4:**
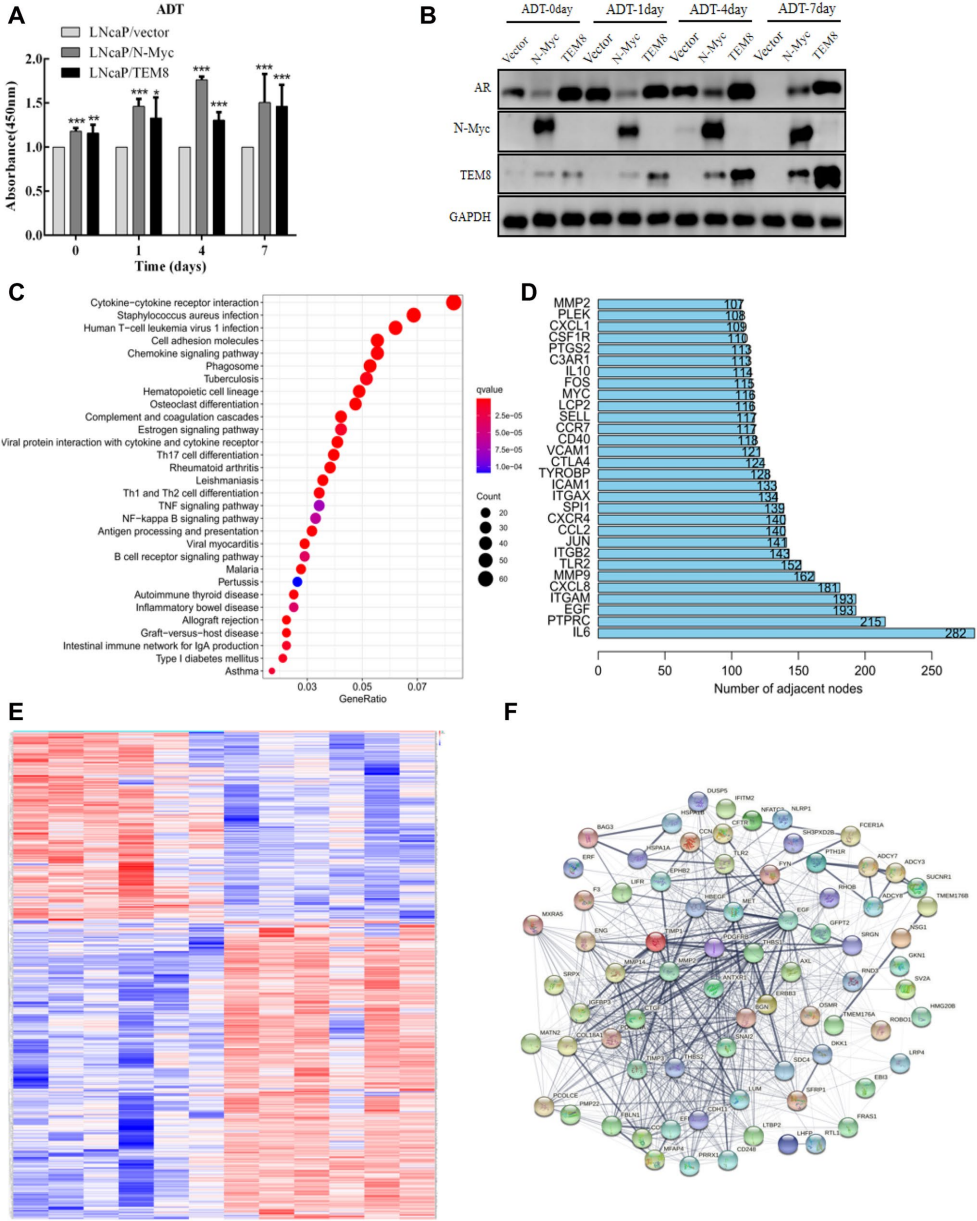
Effects of ADT treatment on proliferation and protein expression in stable cell lines. **A** After 0, 1, 4, and 7 days of ADT treatment, the proliferation rates of LNCaP/*N-Myc* and LNCaP/*TEM8* cells were higher than the control group (*P < 0.05, **P < 0.005, ***P < 0.001). **B** ADT treatment in LNCaP/Vector, LNCaP/*N-Myc*, and LNCaP/*TEM8* cells could not inhibit the expression of *N-Myc* and *TEM8*. **C** KEGG pathway enrichment analysis of the differentially expressed genes after ADT treatment. **D** The Hub genes were identified. **E** A heatmap of ADT-treated differentially expressed genes in the GEO dataset was plotted. **F** Protein–protein interaction (PPI) network related to *TEM8* was constructed using the STRING online database

CCK8 experiment demonstrated that LNCaP/*N-Myc* and LNCaP/*TEM8* cells could promote the proliferation of prostate cancer cells after ADT treatment. This suggests that *N-Myc* and *TEM8* are likely to have ADT resistance (Fig. 4A). With the extension of ADT treatment time, the expression of *AR* protein in LNCaP/Vector cells was decreased. However, the opposite trend was observed in LNCaP/*N-Myc* cells. Although the expression of *AR* did not increase significantly in LNCaP/*TEM8* cells, the expression of *TEM8* was significantly increased (Fig. 4B).

To further confirm our experimental results, we used bioinformatics to analyze differentially expressed genes in PCa before and after ADT. 1693 differentially expressed genes were obtained, including 1227 upregulated and 466 down-regulated genes (Supplementary Table S1). KEGG analysis showed that differential genes were associated with pathways such as cytokine-cytokine receptor interaction, Staphylococcus aureus infection, Human T-cell leukemia virus 1 infection, cell adhesion molecules, and chemokine signaling pathway (Fig. 4C). The network core genes were obtained according to the number of adjacent gene nodes. As shown in (Fig. 4D, Supplementary Table S2), 75 adjacent gene nodes were identified for *TEM8*. The upregulated and down-regulated differential genes were summarized in Fig. 4E. Using the SRTING online database, protein network interacting with TEM8 was constructed (Fig. 4F).

## Discussion

*N-Myc*, a critical oncoprotein required for neuroendocrine tumor development, is overexpressed and amplified in approximately 5% of PCA and 40% of NEPC [16, 17]. During prostate cancer progression, *N-Myc* overexpression can potentiate the escape of tumors from *AR* and promote the development of CRPC and NEPC [8]. Dardenne et al. found that *N-Myc* can cooperate with *EZH2* to establish a new signaling pathway that can drive the differentiation of prostate cancer to neuroendocrine [7]. Emerging evidence shows that *N-Myc* amplification is associated with high vascular density in neuroblastoma [18]. However, the detailed underlying mechanism of action of *N-Myc* in PCa is not fully understood.

*TEM8*, an integrin-like cell-surface transmembrane protein, is highly upregulated in the tumor endothelium and expressed in several cancer types [19]. Antibodies against *TEM8* have shown a broad range of anti-tumor activity due to their ability to target *TEM8* and selectively inhibit pathological angiogenesis without causing severe side effects [20]. In breast cancer, specific antibodies against *TEM8* can target cancer stem cells and tumor-associated vascular systems to inhibit tumor progression [21]. Although these suggest that *TEM8* can be a therapeutic target, such evidence remains unknown in prostate cancer.

We found that the expression of *N-Myc* and *TEM8* in clinical samples correlated with prostate cancer tissue type, tumor progression, and patient’s prognosis. The positive rates of *N-Myc* and *TEM8* were significantly associated with a high Gleason score (Gleason score above 7) and TNM stage (III/IV). Additionally, *N-Myc* and *TEM8* were significantly higher in patients with bone metastasis than those without bone metastasis. Furthermore, expressions of *N-Myc* and *TEM8* were associated with poor prognosis in prostate cancer patients. These results suggest that *N-Myc* is a predictor of advanced stage of prostate cancer and plays a crucial role in prostate cancer progression.

Moreover, we demonstrated a significant positive correlation between *N-Myc* and *TEM8* expression in PCa samples. Further experiments revealed that overexpression of *N-Myc* upregulated the expression of *TEM8 in* prostate cancer cells*.* This finding is consistent with previous report by Dardenne et al. [7]. Furthermore, our results showed that *N-Myc* promoted the proliferation rate of prostate cancer cells by regulating *TEM8*. However, the regulatory action of *N-Myc* on *TEM8* in prostate cancer still requires further exploration.

It has been demonstrated that the incidence of lethal PCa might increase following the increased usage of androgen deprivation therapy, which has brought difficulty in treating patients with advanced prostate cancer. Our tubule formation assays confirmed that *N-Myc* and *TEM8* may promote angiogenesis in prostate cancer cells. Furthermore, we found that overexpression of *N-Myc* and *TEM8* significantly increased the proliferation of PCa cells after ADT treatment. However, compared with the control group, the expression level of *AR* protein in *N-Myc* overexpression group was not inhibited but showed a gradual increase. Consistent with our findings, previous studies have demonstrated that *N-Myc* has an inhibitory action on the expression of *AR* in PCa cells, thus rendering ADT ineffective. We showed that *N-Myc* upregulation after ADT treatment was associated with an increase in the expression of *TEM8*. Interestingly, *AR* protein levels in cell lines overexpressing TEM8 did not change significantly after ADT treatment, suggesting that *TEM8* can potentiate the escape of prostate cancers from castration. This is suggestive that *N-Myc* can potentiate the escape of prostate cancer cells from ADT therapy by upregulating the expression of *TEM8*.

Changes in protein levels after ADT and tubule formation assays in our study showed that *N-Myc* might increase therapeutic resistance and angiogenesis in prostate cancer by regulating *TEM8*.

## Conclusion

**Fig. 5:**
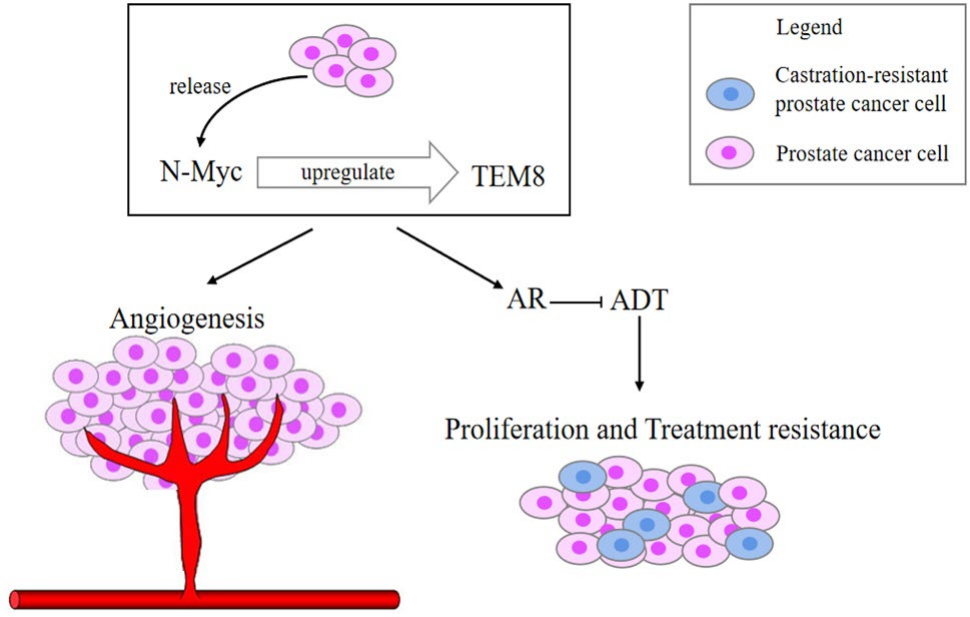
Proposed mechanism of how *N-Myc* and *TEM8* promote angiogenesis and therapeutic resistance of prostate cancer. *N-Myc* can regulate the expression of *TEM8* in prostate cancer tissues. In prostate cancer cells, both *N-Myc* and *TEM8* can induce AR which makes prostate cancer cells resistant to ADT therapy. Further, both *N-Myc* and *TEM8* can promote angiogenesis and the proliferation of prostate cancer cells

Our study has revealed that *N-Myc* and *TEM8* can promote angiogenesis and therapeutic resistance in prostate cancer (Fig. 5). This study also showed for the first time that *N-Myc* could regulate the expression of *TEM8* in prostate cancer. We also demonstrate that *TEM8* is associated with markers of prostate cancer progression. Hence, our finding supports the notion that *TEM8* can be used as a marker to indicate treatment response in patients with advanced prostate cancer.

## Supplementary Information

Below is the link to the electronic supplementary material.

Supplementary file1 (et 223 kb)

Supplementary file1 (et 109 kb)

## Data Availability

The datasets used in this study are available from the first author upon reasonable request.

## Abbreviations

CRPC: Castration resistance prostate cancer
ADT: Androgen deprivation therapy
BPH: Benign prostatic hyperplasia
PCa: Prostate cancer
IHC: Immunohistochemistry
HUVEC: Human umbilical vein endothelial cell
PIN: Prostate intraepithelial neoplasia
shRNA: Short hairpin RNA
AR: Androgen receptor
DEGs: Differentially expressed genes
PPI: Protein–protein interaction
NEPC: Neuroendocrine prostate cancer
